# Going to extremes: progress in exploring new environments for novel antibiotics

**DOI:** 10.1038/s44259-024-00025-8

**Published:** 2024-03-25

**Authors:** Gerry A. Quinn, Paul J. Dyson

**Affiliations:** 1 Centre for Molecular Biosciences, Ulster University, Coleraine, BT52 1SA, N Ireland UK; 2 Institute of Life Sciences, Medical School, Swansea University, Singleton Park, Swansea, SA2 8PP Wales UK

**Keywords:** Antibiotics, Antimicrobial resistance

## Abstract

The discoveries of penicillin and streptomycin were pivotal for infection control with the knowledge subsequently being used to enable the discovery of many other antibiotics currently used in clinical practice. These valuable compounds are generally derived from mesophilic soil microorganisms, predominantly *Streptomyces* species. Unfortunately, problems with the replication of results suggested that this discovery strategy was no longer viable, motivating a switch to combinatorial chemistry in conjunction with existing screening programmes to derive new antimicrobials. However, the chemical space occupied by these synthetic products is vastly reduced compared to those of natural products. More recent approaches such as using artificial intelligence to ‘design’ synthetic ligands to dock with molecular targets suggest that chemical synthesis is still a promising option for discovery. It is important to employ diverse discovery strategies to combat the worrying increase in antimicrobial resistance (AMR). Here, we reconsider whether nature can supply innovative solutions to recalcitrant infections. Specifically, we assess progress in identifying novel antibiotic-producing organisms from extreme and unusual environments. Many of these organisms have adapted physiologies which often means they produce different repertoires of bioactive metabolites compared to their mesophilic counterparts, including antibiotics. In addition, we examine insights into the regulation of extremotolerant bacterial physiologies that can be harnessed to increase the production of clinically important antibiotics and stimulate the synthesis of new antibiotics in mesophilic microorganisms. Finally, we comment on the insights provided by combinatorial approaches to the treatment of infectious diseases that might enhance the efficacy of antibiotics and reduce the development of AMR.

## Introduction

Physicians in the early 20^th^ century employed various chemical agents such as mercury salts and sulpha drugs to treat pathogenic microbial infections, however, many of these were also associated with problems of toxicity or poor efficacy^1^. A fortuitous breakthrough came in 1928 with the discovery of penicillin by Dr Alexander Fleming, and subsequently streptomycin from a Gram-positive *Streptomyces* bacterium by Schatz and Waksman in 1943^2,3^. These antibiotics are produced by a filamentous fungus and a Gram-positive *Streptomyces* bacterium, respectively. Selman Waksman coined the term antibiotic to describe compounds of biological origin that can kill or inhibit the growth of microorganisms^4^. Although this definition has changed slightly in the intervening period, antibiotics have revolutionised clinical care and gone on to be one of the greatest medical success stories of the 20^th^ century. This progress was significantly aided by governments and a collective of pharmaceutical companies who ensured the mass production and distribution of these life-saving drugs in those early years which were commonly referred to as the beginning of the ‘golden era of antibiotic discovery’^1^.

Inspired by Waksman’s pioneering research, scientists went on to discover and isolate many antibiotic-producing soil microorganisms. Although these antibiotics now comprise approximately 70% of our current frontline antibiotics, this discovery process trickled to a halt in the 1970s when scientists realised that they were (re)discovering the same compounds over and over again in what was described as a phenomenon of replication^5^. The pharmaceutical industry tried to compensate for this gap in the discovery pipeline by using combinatorial chemistry, however, even though millions of compounds were tested in these screening programmes, very few progressed to further developmental stages due to problems with toxicity, stability, and overall efficacy^6^. In comparison, the success rate for the development of drugs from natural products is about 0.6%, which is approximately 100 times greater than that of synthetic compounds^7^. This is in part due to the realisation that novel synthetic molecules occupy a much smaller chemical space in terms of their underlying chemical structures in comparison to natural products, and has motivated other efforts focused on combinatorial biosynthesis^8^. Essentially this process offers the prospect of redesigning known antibiotic structures to create new activities based on knowledge of biosynthetic pathways. This approach has been partly hindered by low yields, but it was also acknowledged that as the resulting compounds share a core chemical scaffold with their natural counterparts, resistance would arise quickly. Consequently, with a low return on the investment for pharmaceutical companies associated with these programmes, there has been a scaling down of antibiotic R&D investment by the pharmaceutical industry^9^, unfortunately coinciding with increasing problems with AMR.

More recently, artificial intelligence has been exploited, in place of traditional screening of chemical libraries, to discover new compounds that inhibit bacterial growth^10^. This is an encouraging advance, as it underlines the importance of accessing chemical diversity, be it sourced via synthesis or through natural products. Still, the adoption of next-generation genome sequencing with genome mining, combined with a wider exploration of natural habitats and a deeper understanding of the biology of antibiotic-producing microorganisms, suggests that only a small fraction of natural products with potential antibiotic activity have been examined to date. Indeed, mathematical modelling of the potential for antibiotic biosynthesis by the genus *Streptomyces* has predicted that they could produce up to 100,000 different antibiotics, of which only a small fraction have been discovered to date^11^. This review focuses on the exploration of extreme and unusual habitats for new antibiotic-producing organisms, with emphasis on actinobacteria and, in particular, the most prolific producers that belong to the genus *Streptomyces*. Rather than provide an exhaustive view of all antimicrobials sourced from these extreme and unusual environments, we provide an overview and reference key detailed reports where further information can be gleaned.

## The biology of antibiotic-producing microorganisms

Two of the main producers of antibiotics, filamentous fungi and actinobacteria, typically share similar life-styles, inhabiting soil and/or marine sediments. They are often non-motile, grow as branched mycelium, adopt a saprophytic lifestyle and many produce reproductive spores. However, much of what we know concerning the biology and synthesis of antibiotics has been derived from a rather limited study of cultures of individual microbial species grown under laboratory conditions rather than examining in situ the physiology of antibiotic synthesis.

Antibiotics are the products of secondary metabolism whereby microorganisms can repurpose their metabolites from primary metabolism to generate often quite complex organic compounds using a dedicated set of enzymes for each secondary metabolite they produce. The specific enzymes required for antibiotic biosynthesis of any given secondary metabolite are encoded on a set of genes organised in a biosynthetic gene cluster (BGC), permitting coordination of their expression. Studies in *Streptomyces* indicate that this antibiotic production often coincides with reproductive growth when elements of the mycelium are recycled to fuel the growth of spore-bearing aerial hyphae^12^. In this context, antibiotics may provide a defence against predation by other competing organisms. However, there is some debate about the ecological role of these compounds and whether sufficient amounts (of antibiotics) are produced in natural environments to function in antibiosis. This may be due to the fact that antibiotics can have many physiological roles including acting as potent signalling molecules at lower concentrations^13^. Further, each antibiotic-producing organism can simultaneously express several secondary metabolites which may act in a synergistic manner^14^. Genome sequencing has also revealed that, in addition to the BGCs that are constitutively expressed and whose products can be determined, there are also many BGCs that are ‘cryptic’ or ‘silent’, for which no end-product can be determined^15^. This may be a consequence of growing pure cultures in lab conditions, which cannot fully recreate the complexities of the natural environment. In addition, it should be noted that genome annotation algorithms such as antiSMASH^16^, which are used for the identification of BGCs in a genome sequence, are trained in reference to known BGCs. One of the consequences of this is that entirely novel BGCs, with the potential to direct the synthesis of a new class of antibiotic, could theoretically be overlooked.

One increasing area of study, albeit as yet limited in breadth, is investigating how antibiotic-producing microbes behave under in situ conditions—a critical issue with respect to understanding the cues that may trigger antibiotic biosynthesis, particularly by cryptic pathways. For example, terrestrial antibiotic-producing microorganisms such as *Streptomyces* spp. are frequently associated with the soil rhizosphere and can provide growth advantages to plants in return for physiologically favourable growth conditions^17^. Indeed, elegant studies have shown how leaf-cutter ants exploit biosynthesis of antifungal compounds using symbiotic actinobacteria to protect their ‘gardens’ of a cellulose-digesting fungus from competing fungi^18^. Evidently, these types of interactions vary considerably according to the local fauna and flora specific to any given habitat, particularly in the case of extreme environments. Together with the challenges posed by adaptations to physiologically extreme or unusual environments, the specific conditions encountered by extremophilic, extremotolerant or other microorganisms have likely provided an evolutionary pressure to diversify their antibiotic production capacity. Based on this rationale and with the global AMR crisis as a key driver, bioprospecting for new antibiotics sourced from previously untapped extreme and unusual environments is of vital importance.

For those organisms isolated from inhospitable habitats, the types of microorganisms to consider are those associated with long periods of extreme temperatures, such as extremes of heat (thermophiles/thermotolerant) or close to freezing (psychrophiles/psychrotolerant); habitats with extreme pressures, like high plateaus or deep ocean trenches (barophiles/barotolarant); habitats with extreme osmotic pressure, such as salt plains (halophiles/halotolerant); habitats with extreme pH, such as acid (acidophiles/acidotolerant) or alkali (alkaliphiles/alkalitolerant); and habitats with extreme arid conditions (xerophiles/xerotolerant), such as the desert. More than one of these conditions may persist in many habitats, and consequently, the succeeding sections in this review are at best arbitrary. The related scientific literature frequently alludes to antibiotics produced by extremophiles; however, we note that for many examples these isolated organisms are better described as having extremotolerant characteristics.

It should also be noted that in many cases, especially with respect to the streptomycetes, bacterial systematics has depended heavily on 16s rDNA sequence comparisons and, prior to this, chemical taxonomy. Access to next-generation sequencing has now enabled multi-locus sequencing or whole genome comparisons, providing a more reliable means for the speciation of members of the genus, albeit applied so far to only a small proportion of isolated species.

## Antibiotics from thermotolerant producers

Desert ecosystems have attracted considerable attention from microbiologists due to the discovery of microorganisms that live in seemingly uninhabitable temperatures which at their peak can reach as high as 56.7 °C (July 10, 1913, Death Valley, California) in the daytime and −3.9 °C in the night-time. Microorganisms are able to survive in such inhospitable conditions by physiological adaptations and/or associations with other organisms. For example, many deserts and drylands have a biological soil crust on top of the sand which is formed primarily by the adhesion of soil particles to extracellular polysaccharides excreted mainly by cyanobacteria^19^. These biofilm mats also include a complex microbial consortium consisting of fungi, algae and occasionally lichens and are vital for creating and maintaining the fertility of this layer by fixing both carbon and nitrogen^19^. It is in these environments that researchers have identified many extremophiles^20^. One important area for these discoveries is the Atacama desert in Chile^21^. This is a large desert plateau on the Pacific coast of Chile which covers approximately 100,000 km^2^ and whose core region has been described as too extreme for life^22^. Researchers have identified at least 50 novel natural products from this source alone, many with antibiotic activity^20^. These bacteria can also be considered to be xerotolerant and/or halotolerant, that is, organisms that can survive in very arid conditions or those with high salt stress^23^. Other important sources of antibiotic-producing desert extremophiles have been reported for the Sahara desert in North Africa (9.2 million km²), the Taklamakan Desert in China (337,000 km^2^) and the Thar desert in India and Pakistan (200,000 km^2^)^23^ (Table 1).

**Table 1:** Examples of novel antibiotics and their thermotolerant producers

Antibiotic	Organism	Origin	Activity against	Ref
Brasiliquinone E	*Nocardia* sp. XJ31	Xinjiang, China	*Mycobacterium tuberculosis*	^72^
Dithiolopyrrolone PR11	*Saccharothrix algeriensis* NRRL B-24137	Sahara, Algeria	Gram-positive bacteria, fungi and yeasts	^73^
Asenjonamides A-C	*S. asenjonii* KNN 42.f	Atacama desert, Chile	*S. aureus*, *B. subtilis*, *E. coli*, *E. faecalis*, and *Mycobacterium smegmatis*	^74^
Chaxalactins A-C	*S. leeuwenhoekii* C34T	Atacama desert, Chile	*S. aureus*, *L. monocytogenes*, and *B. subtilis*, weak activity against *E. coli* and *Vibrio parahaemolyticus*	^75^
Chaxamycins A-D	*Streptomyces* sp. strain C34	Atacama desert, Chile	*S. aureus*, *E. coli*, (epidemic MRSA and Scottish MRSA)	^76^
Atacamycins A-C	*Streptomyces* sp. C38	Atacama desert, Chile	Broad-spectrum antibacterial	^77^
Abenquines A-D,	*Streptomyces* sp. DB634,	Atacama desert, Chile	slightly inhibiting *B. subtilis*	^78^
4-(4-hydroxyphenoxy) butan-2-one	*Streptomyces* sp. TK-VL_333	Southwest Algeria	Antibacterial, antifungal	^79^
Acetic acid-2-hydroxy-6-(3-oxo-butyl)-phenyl ester	*Streptomyces* sp. TK-VL_333	Southwest, Algeria	Antibacterial, antifungal	^79^
New anthracycline glycoside	*Streptomyces* sp. SAS09	Thar desert, India	Antibacterial	^80^

## Antibiotics from psychrotolerant producers

Antibiotics have also been discovered in psychrotolerant microorganisms. These organisms are able to survive freezing temperatures and repeated heat-thaw cycles and are generally found in cold environments such as polar regions, cold deserts, glaciers, deep oceans and vast areas of permafrost. Although these microorganisms may be better known for having cryoprotective compounds, they have also produced novel antibiotics^24–27^. Many of these discoveries were made in dedicated research facilities such as those in the Arctic region^25^, the Barents sea^27^, Svalbard in the Norwegian archipelago^24^ and the Antarctica^28^ (Table 2). A good summary of many of these discoveries from Antarctic is provided in a review by Núñez-Montero et al.^28^.

**Table 2:** Novel antibiotics from psychrotolerant microorganisms

Antibiotic	Organism	Origin	Activity against	Ref
Lindgomycin	Fungus of *Lindgomycetaceae*	Antarctic	MRSA	^26^
Arcticoside	*Streptomyces* sp	Arctic marine	*Candida albicans*	^25^
Bisvertinolone	*Aspergillus protuberus* MUT3638	Barents Sea, Northern Europe	*Staphylococcus aureus*	^27^
2-amino-3-dodecanol	*S. avidinii* SB9	Permafrost, Norway	Gram-positive and negative bacteria, *Candida*	^24^

## Antibiotics from aquatic environments

Considerable attention has also been devoted to aquatic environments in the search for new antibiotics^29^. These ecosystems are easily some of the largest in the world and are the source of many antibiotic-producing organisms including halophiles, barophiles, and thermophiles. A recent review of the progress in this area between 1984 and 2022, indicated that 182 natural products were derived from predominantly filamentous fungi and *Streptomyces* species growing in these extreme conditions; half of these being novel compounds with antibiotic activity^30^. We have compiled a list of some of these antibiotics in Table 3. For *Actinobacteria*, 70% of antimicrobial compounds were sourced from bacteria isolated from marine sediment and 24% were isolated from bacteria associated with marine flora and fauna^31^. Amongst this later group, algae, sponges, corals and mangroves were the most common environments to isolate antibiotic-producing fungi and actinobacteria^32^ (Table 4). One feature of antibiotics sourced from the marine environment is that they generally possess a greater abundance of halogen-containing compounds in comparison to those obtained from terrestrial organisms, reflecting the halogenated nature of seawater.

**Table 3:** Examples of novel antibiotics isolated from marine producers

Antibiotic	Organism	Origin	Activity against	Ref
Ophiobolin sesterterpenoid	*Aspergillus insuetus* SD-512	Cold seep, South China Sea	Broad-spectrum antibacterial	^81^
Compound 1	*Streptomyces* sp. WU20	Hydrothermal vent Kueishantao, Taiwan	*Bacillus subtilis*	^35^
New dixiamycins	*Streptomyces olivaceus* OUCLQ19-3	Cold seep, South China Sea	Multi-drug-resistant strains	^82^
Asperoxide A	*Aspergillus nidulans* SD-531	Cold seep, South China Sea	Several Gm-negative bacterial species	^83^
Marthiapeptide A	*Marinactinospora thermotolerans* SCSIO 00652	South China Sea sediment	Gm-positive bacteria	^84^
Desotamide B	*S. scopuliridis* SCSIO ZJ46	South China Sea sediment	Gm-positive bacteria	^85^
Phocoenamicins B and C	*Micromonospora sp*.	Marine sediment, Canary Islands	MRSA, *M.tuberculosis*	^86^

**Table 4:** Examples of novel antibiotics sourced from microorganisms growing in association with other marine life

Antibiotic	Organism	Origin	Activity against	Ref
6-hydroxymethyl-1-phenazine-carboxamide	*Brevibacterium* sp. KMD 003	*Callyspongia* sp. (Kyeongpo, Gangwondo, Korea)	*Enterococcus hirae* and *Micrococcus luteus*	^87^
1,6-phenazinedimethanol	*Brevibacterium* sp. KMD 003	*Callyspongia* sp. (Kyeongpo, Gangwondo, Korea)	*Enterococcus hirae* and *Micrococcus luteus*	^87^
Monacyclinone F	*Streptomyces sp*. M7_15	Puerto Rican Sponge *Scopalina ruetzleri*	Gm-positive bacteria	^88^
Fridamycin H	Actinokineospora spheciospongiae strain EG49.	*Spheciospongia vagabunda*, (Red Sea, Egypt)	*Trypanosoma brucei*	^89^
Ageloline A	*Streptomyces* sp. SBT345	Mediterranean sponge *Agelas oroides*	*Chlamydia trachomatis*	^90^

There are also many discoveries of antibiotic-producing organisms originating from cold seeps and hydrothermal vents. Cold seeps typically occur over fissures in the ocean floor in environments that are usually the result of tectonic activity. These areas are typically characterised by the seepage of either methane or other hydrocarbons possibly with the addition of hydrogen sulfide. It is this chemically rich environment that sustains the cold-seep ecosystem which is dominated by chemosynthetic primary producers^33^. Contrary to their name, these environments are not necessarily colder than the surrounding water, just cold relative to the hydrothermal vents in their vicinity.

**Fig. 1:**
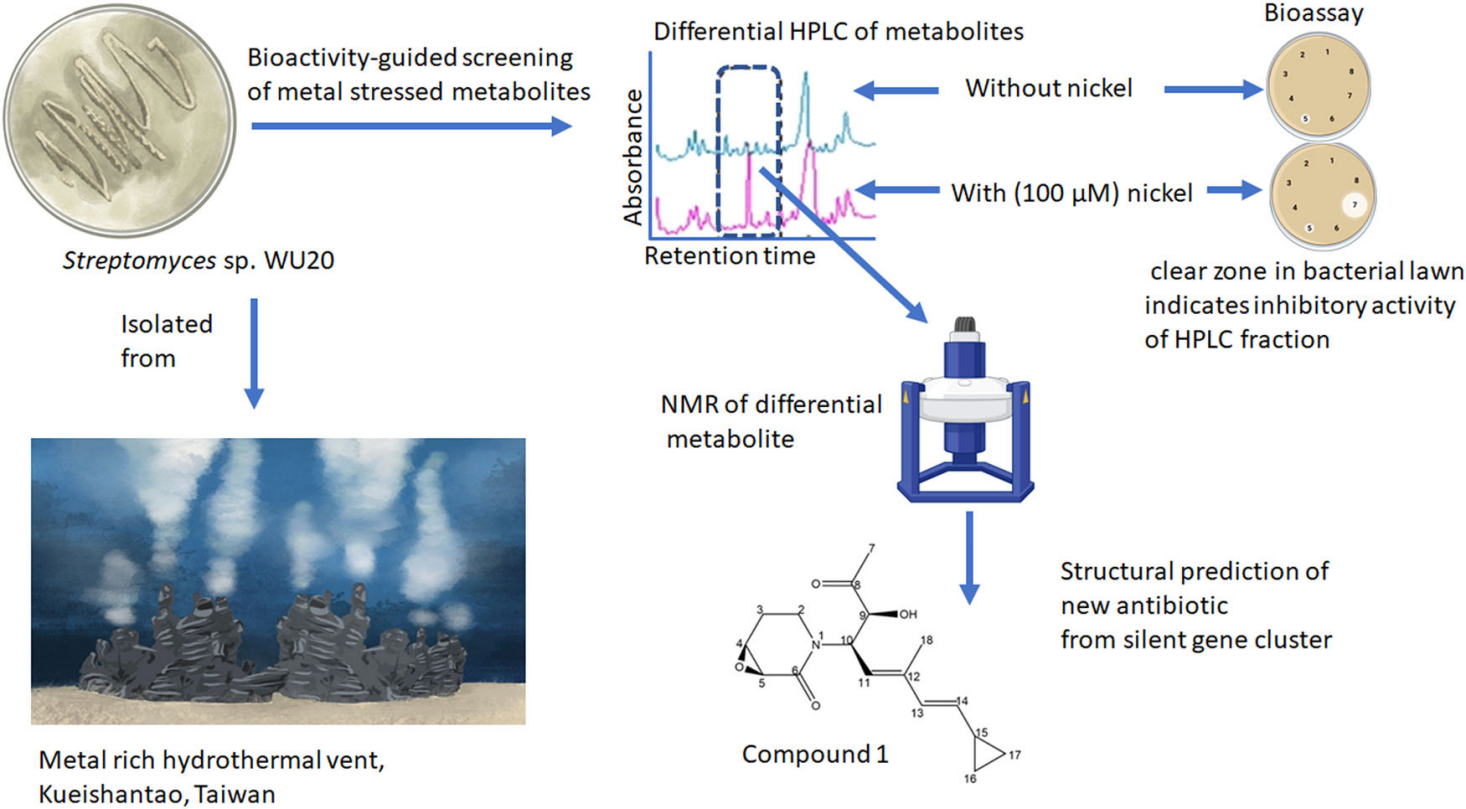
Nickel induction of silent biosynthetic gene clusters in *Streptomyces* sp. WU20 isolated from a metal-rich hydrothermal vent near Taiwan. Bioactive metabolite screening of silent gene induction and analytical metabolomics revealed the structure of a new antibiotic (compound 1). Illustrations of the differential HPLC and the structure of the new antibiotic are adapted from Shi et al.^35^. NMR illustration adapted from “NMR gyrotron” and bioassay illustration adapted from “petri dish antibiotic sensitivity test” both from BioRender.com (2023). Retrieved from https://app.biorender.com/biorender-templates. *Streptomyces* WU20 and hydrothermal vent illustrations by Val Romani.

Hydrothermal vents are also caused by fissures on the sea floor but in this case, superheated water is extruded into the surrounding cold water where dissolved minerals such as iron, copper and zinc are precipitated quickly; many of these precipitates form chimneys which can be colonised by chemosynthetic bacteria^34^. As an example of bioprospecting in such an environment researchers were able to induce the expression of a novel (structurally different) antibiotic produced by *Streptomyces* sp. WU20 follows a strategy based on metal induction of silent biosynthetic gene clusters combined with metabolomic analytical methods. This streptomycete was isolated from a metal-rich hydrothermal vent off the coast of Kueishantao island, which is situated off the coast of Taiwan^35^ (Fig. 1).

## Antibiotics from halophiles

Halophiles or halotolerant microorganisms can grow in a range of salt concentrations, including hypersaline habitats such as the Dead Sea, or the salt flats of the Salar de Atacama^36^. Describing these halophilic/halotolerant antibiotic producers as a separate entity is confounded to a large extent by many of these organisms being associated either with deserts or with the marine environment. Some of the most notable halophilic microorganisms that produce antibiotics can be found in the domain *Archaea*. For example, the archaeocins are ribosomally synthesised bacteriocin-like antimicrobial peptides (AMPs) with antagonistic activity against other microorganisms^37^. Archaeocins fall into two phylogenetic groups: halocins produced by haloarchaea and sulfolobicins produced by the genus *Sulfolobus*. Two novel antibiotic compounds, kribbellichelin A and B, have been isolated from a halophilic actinobacterial *Kribbella* species associated with the rhizosphere of *Limonium majus* which grows in a saline wetland in Spain^38^.

## Antibiotics from unculturable or previously uncultured bacteria

It has been postulated by several scientists that unculturable or uncultivated microorganisms might provide one of the largest sources of new antibiotics, given that more than 99% of an estimated 10^11^–10^12^ microbial species remain undiscovered^39^. One of the solutions to growing unculturable microorganisms may be to conduct the process in situ. This method is exemplified by the iChip^40^ which is seen as a great step forward because it allows for the in situ cultivation of cells in the environment by allowing diffusion of localised nutrients into a chamber^41^. There are also other methods that are currently utilised for uncultivated organisms such as microbial traps or double isolation methods^39^. The use of the iChip led to the discovery of teixobactin, derived from the bacteria *Eleftheria terrae*, after screening 10,000 isolates. Teixobactin has inhibitory activity against Gram-positive bacteria and mycobacteria but not Gram-negative organisms. Its mode of action is the inhibition of peptidoglycan and teichoic acid synthesis by inhibiting lipids^42^. Another antibiotic isolated using the iChip is clovibactin. This also blocks cell wall synthesis by targeting multiple peptidoglycan precursors^43^. Further antibiotic discovery using this method included a macrolide known as amicobactin, which inhibits Mycobacterium tuberculosis, and hypeptin (produced by Lysobacter sp. K5869) which is similar to teixobactin and displays a broad range of activity against Gram-positive bacteria^40^. Bacteria which are described as “just difficult to isolate” have also been seen as a good source of novel compounds. This is exemplified by the research of Rolf Müller’s group^44^ resulting in the discovery a new antibiotic, rowithocin, from *Sorangium cellulosum* which has an uncommon phosphorylated polyketide scaffold^45^. Applying innovative culturing and isolation techniques for extremophilic/extremotolerant microorganisms offers the potential for the discovery of many new antibiotics.

## Antibiotics from traditional and historic medicines

There is growing evidence that the therapeutic basis of some traditional and historic medicines is based on a relationship with antibiotic-producing organisms^46–48^. For example, traces of tetracycline have been found in human skeletal remains of ancient Sudanese Nubia dating back to 350–550 CE^47,48^. This can only be explained if these ancient people were exposed to tetracycline-containing materials in their diets, consistent with a low rate of infectious disease documented in the population^48^. In other research, a milky white exudate covering the rock surfaces of some caves which were referred to in ancient texts as ‘moonmilk’ was used to heal multiple ailments^49^. This moonmilk contains an abundance of *Streptomyces* that have antibacterial activity against a wide range of bacteria and fungi^49,50^. Researchers have isolated *Streptomyces* sp. HZP-2216E from a traditional Chinese medicine that incorporates the sea lettuce Ulva pertusa which is associated with a bacteria that produces a unique indolizinium alkaloid, streptopertusacin A and two previously undescribed bafilomycins with antibiotic activity^46^.

Another common theme in the discovery of inhibitory compounds from traditional and historic medicines is their association with particular soils; however, these tend to have unique characteristics that differentiate them from other soil types such as higher pH or the presence of specific minerals^51–53^. These soils have been documented in Jordan where a red soil known to contain actinobacteria that produce actinomycin is traditionally used to treat skin infections^51^, in northern British Columbia in Canada, where generations of indigenous people have used Kisameet clay (glacial clay) that has potent activity against many important clinical pathogens^53^ and in Northern Ireland, where researchers isolated *Streptomyces sp*. myrophorea, from an ancient Irish folk cure which was based on soil from a post-glacial alkaline grassland soil which has in-vitro antibiotic activity against clinically important ESKAPE pathogens^52^. Indeed subsequent investigations of a similar soil from the immediate vicinity identified many more *Streptomyces* with antibacterial and antifungal activity^54^.

These shared associations of traditional and historic medicines with antibiotic-producing bacteria^52,53^ open the door to the possibility that many more discoveries could be made using a more systematic approach. However, this discussion would be incomplete, without at least crediting the pioneering work in this field by Geoffrey Cordell even though his focus was anticancer medicines from plants. In his research, Dr Cordell observed that the screening of historic and traditional medicines yielded a far greater positive rate than the random screening of plants^55^.

## Combinatorial approaches to antimicrobial therapy

One increasingly relevant factor missing from discussions of traditional and historic medicines is that most of these are rarely purified compounds but instead a combination of ingredients^56,57^. In many instances, researchers are also uncertain which particular ingredients are strictly necessary to produce maximum efficacy^56^. So why is this relevant to treating pathogens resistant to conventional therapies or even delaying the onset of AMR? The principal antibiotic producers such as *Streptomyces* spp. typically produce a cocktail of secondary metabolites that can exhibit complementary antimicrobial activity. For instance, many antibiotic-producing organisms produce biosurfactants that can have synergy with antibiotics. One good example is the combination of sophorolipids (biosurfactant) with tetracycline, which increases the inhibitory activity against *Staphylococcus aureus* by 25%. Further trials with Gram-negative bacteria (*E. coli*) demonstrated that sophorolipids combined with cefalor increased inhibitory activity by 48% more than cefalor alone^58^. In some cases, the biosurfactants have weak antibacterial activity on their own, for instance, lipopeptides from *Streptomyces rochei* have antagonistic properties against *Staphylococcus aureus* and *Pseudomonas aeruginosa*^59^. The role of co-produced antibiotics has been reviewed in some detail by Meyer and Nodwell^14^ who noted that biosynthetic genes directing synthesis of complementary compounds are often located adjacent to each other on BGC superclusters. Of course, combining antibiotics is not a new clinical practice and has been used for many years in the treatment of tuberculosis for fear of resistance arising^60^. However as noted earlier, there are other compounds with no apparent antibiotic activity on their own that can have synergistic effects with antibiotics^61^. This is demonstrated in the case of a reducing agent, alkylresorcinol which in combination with antibiotics such as gentamicin, polymyxin, ampicillin and vancomycin inhibit various pathogenic bacteria^62^. Perhaps a more well-known example of these synergies is siderophores which are iron scavenging compounds commonly produced by actinomycetes. These can act as useful adjuvants to antibiotics. For example, desferrioxamine, produced by *Streptomyces pilosus* has synergistic activity with gentamicin, chloramphenicol, cefalothin, cefotiam and cefsulodin against pathogenic bacteria^63^. Siderophores are also found naturally in combination with antibiotics in the form of sideromycins^64,65^. These include albomycins, which are combination of a thioribosyl nucleoside linked to a ferrichrome-type siderophore and have antibacterial activity against Gram-positive and Gram-negative bacteria^66^ and ferrimycins which are iron-containing siderophores with activity against Gram-positive bacteria^65,67^.

Even secondary metabolites as seemingly innocuous as pigments can sometimes have some synergistic activity. This is demonstrated in research on a green pigment produced by the marine bacteria *Streptomyces tunisiensis* W4 which has a synergistic inhibitory activity when combined with cefuroxime and ciprofloxacin^68^. In addition, some pigments also have antibiotic activity on their own such as undecylprodigiosin, a red pigment produced by *Streptomyces* sp. JAR6 which has been noted for its antibiotic activity against *Salmonella* sp., *Proteus mirabilis*, *Shigella* sp. and *Enterococcus* sp,^69^.

**Fig. 2:**
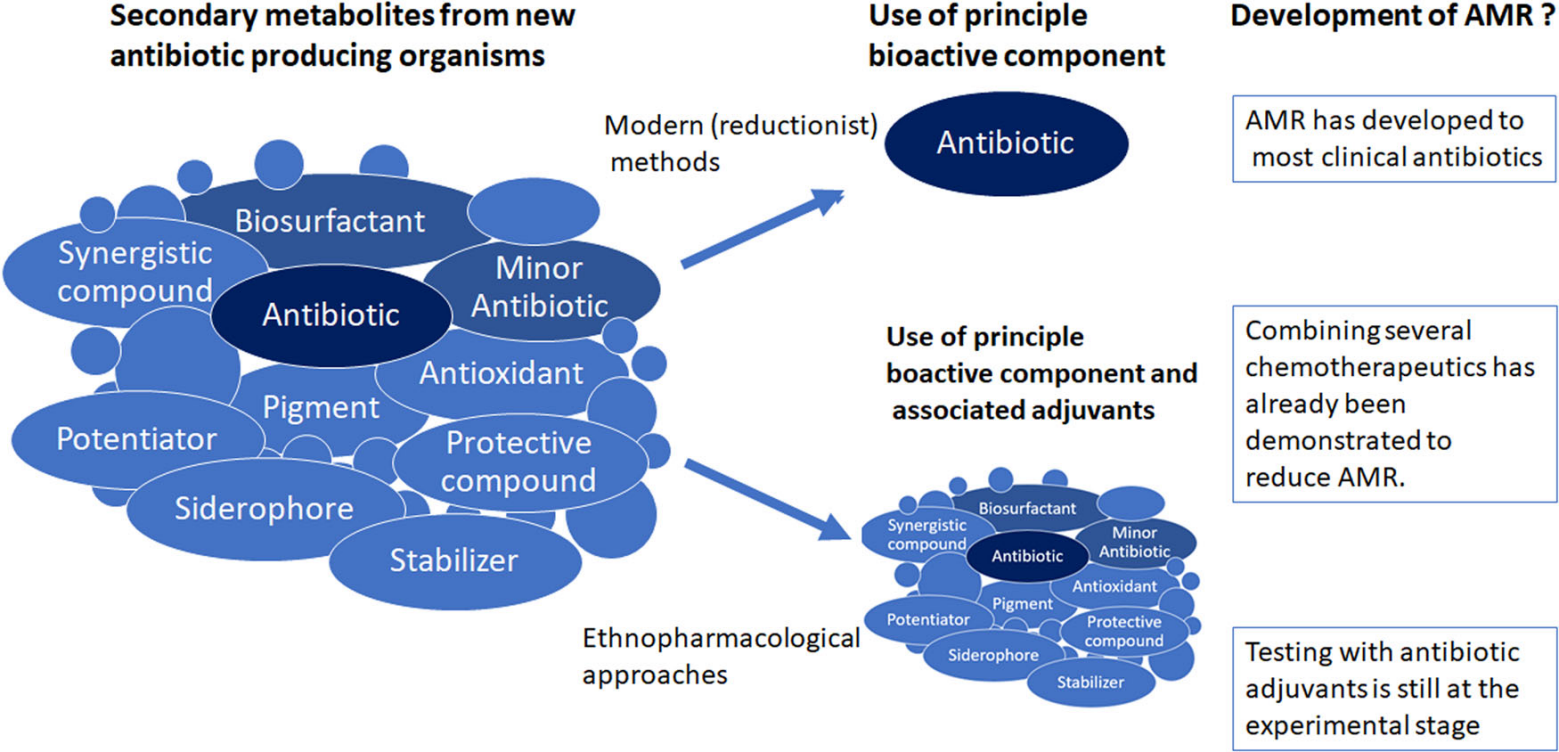
Infectious disease treatment strategies using classical reductionist and combinatorial approaches. Antibiotic-producing organisms produce a wide array of secondary metabolites. In modern reductionist approaches, the principle antibiotic component is purified and used to treat infections. In combinatorial approaches, various other secondary metabolites may be combined with the principle antibiotic to create a synergistic action.

Many of these synergies could potentially enhance the therapeutic activity of antibiotics allowing for a reduced dosage and potentially reducing the development of AMR^61^ (Fig. 2).

## Exploiting insights learned through isolation and identification of extreme or unusual antibiotic-producing organisms

**Fig. 3:**
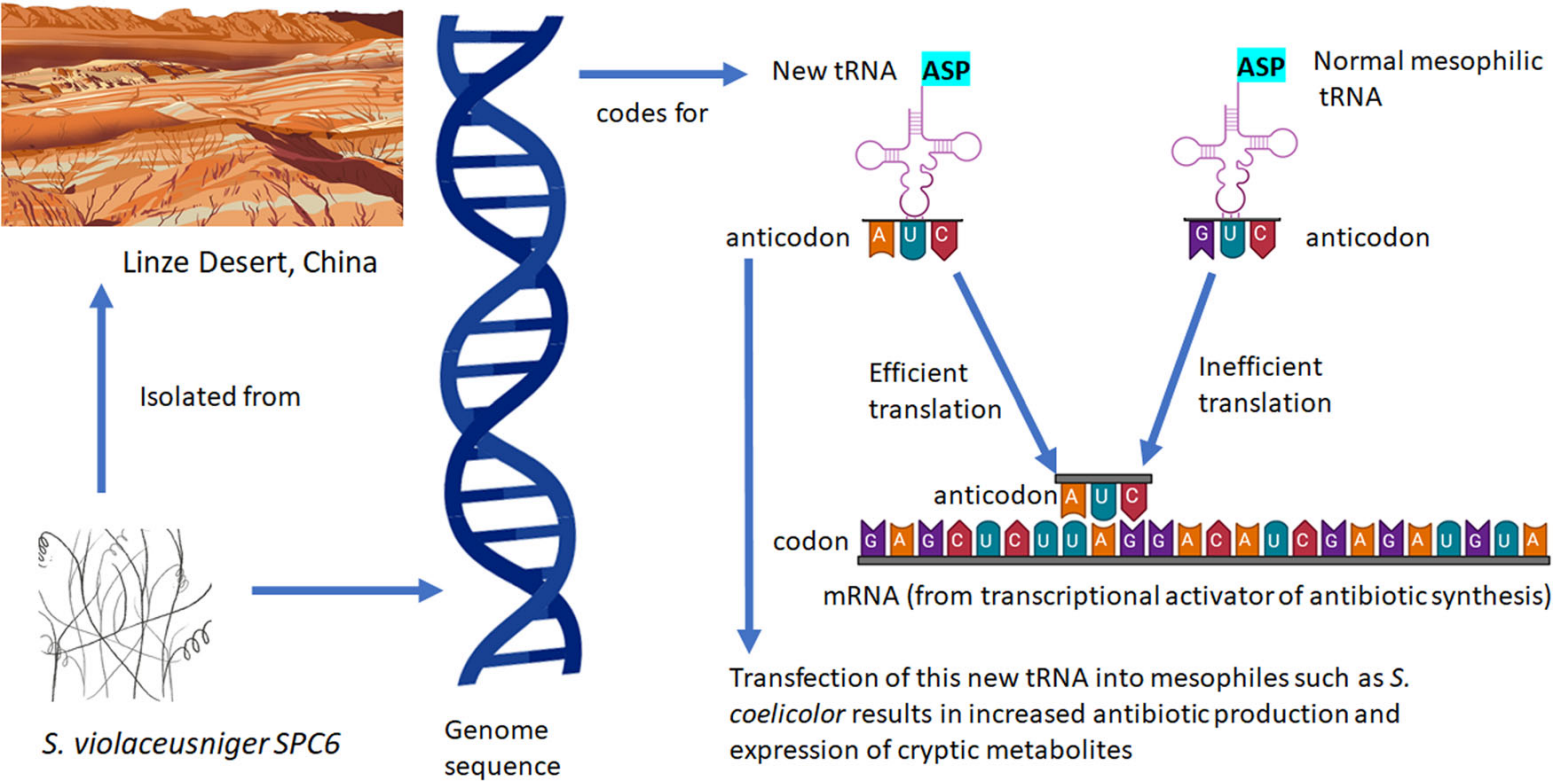
A new tRNA from *S. violaceusniger* SPC6 circumvents inefficient wobble base-pairing during translation. The new tRNA-Asp-AUC is responsible for the efficient translation of GAU codons in *S. violaceusniger* SPC6. In the absence of queuosine tRNA anticodon modification, the new tRNA circumvents inefficient wobble base-pairing during translation. When the tRNA is transfected into model mesophilic species *S. coelicolor*, it greatly enhances synthesis of 4 different antibiotics including the product of a so-called cryptic pathway^71^. Illustrations from Biorender.com (2023) include NMR, adapted from “NMR gyrotron”, bioassay, adapted from “petri dish antibiotic sensitivity test”, and genome sequence, adapted from “modification of group icon, “DNA transfection (step 4)”. Retrieved from https://app.biorender.com/biorender-templates. Other illustrations of *S. violaceuniger* and the Linze Desert were by Val Romani.

It is generally expected that extremophilic or extremotolerant microorganisms display substantial differences in physiology compared to their mesophilic counterparts. Analysis of these physiological differences could also play a role in elucidating new methods for triggering antibiotic synthesis. Although this is a largely unresearched area, one recent study is encouraging in this context. *Streptomyces violaceusniger* strain SPC6, a halotolerant strain with an optimal growth temperature of 37 ^o^C, was isolated from the Linze desert in China^70^. Remarkably this streptomycete completes its life cycle very rapidly, sporulating within 2 days, in contrast to the 4 or 5 days required for typical mesophilic streptomycetes that grow optimally at 28 ^o^C. Sequencing of this SPC6 genome revealed a novel tRNA gene encoding tRNA-Asp-AUC^71^. Its cognate GAT codon is over-represented in both pleiotropic and pathway-specific transcriptional activators of antibiotic biosynthesis in streptomycetes and translation of this codon in mesophiles is normally dependent on inefficient wobble base-pairing by the conserved tRNA-Asp-GUC. Expression of the new tRNA in mesophilic producers of commercial antibiotics resulted in precocious overproduction of these antibiotics. Also of note is that the new tRNA activated the expression of a cryptic BGC in *S. coelicolor*, resulting in the production of the antibiotic coelimycin (Fig. 3)^71^. While it is too early to predict whether this can be a generic tool for activating silent BGCs, it is nonetheless a key advance in unlocking the biosynthetic potential of the actinobacteria and could help in the discovery of new antibiotics in the future.

## Conclusions

Bioprospecting for new antibiotic-producing microorganisms in extreme and unusual environments has been a very fruitful exercise, underlined by the novelty of many of the resulting new antimicrobials discovered. One criticism often levelled at this type of exploration is that it has in most cases yielded new activities based on known chemical scaffolds, rather than new classes of compounds. However, these new antibiotics often have potent activities against pathogens resistant to current frontline antibiotics, such as MRSA. An implication is that evolution has finessed new antibiotic activities based on a limited set of optimal chemical scaffolds and begs the question of whether we are misguided in the belief that a solution to AMR depends on the discovery of novel antibiotic classes. Taking forward any of the new antibiotics discovered from producers isolated from extreme environments for clinical trials would require a large investment from the pharmaceutical industry. However, one likely barrier to this investment is the possibility of a repeat of the cycle of emerging resistance that would devalue any new drug. But the success of antibiotic-producing microorganisms in their natural environments suggests that we can learn more from these producers themselves and formulate combinations of antibiotics or antibiotics with adjuvants that could help to increase antibiotic efficacy and minimise resistance. It is now almost a century since Fleming’s discovery of penicillin revolutionised medical practise. Since then, the traditional reductionist approach has been one of isolating an antibiotic-producing organism, growing this in a large-scale monoculture, and purifying the active component. This manner of production and the subsequent prescription of a single antibiotic may be easier in the short-term for health-care regulators to evaluate, but we can now see the long-term (albeit less than 100 years) cost of this approach in terms of lives lost to untreatable infectious diseases due to AMR. Quite possibly, solutions are already at hand, including the contribution of new antibiotics discovered from producers isolated from extreme environments, but we need to be more judicious in how we apply these advances for the benefit of mankind in the future. The key to this is to identify synergies between antibiotic compounds and thereby derive formulations that mitigate against the development of AMR, safeguarding these valuable medicines for the future.

## Data Availability

All data generated or analysed during this study are included in this published article.

## References

[1] Aminov, R. I. A brief history of the antibiotic era: lessons learned and challenges for the future. *Front. Microbiol.* **1**, 134 (2010). 21687759 10.3389/fmicb.2010.00134 PMC3109405

[2] Fleming, A. On the antibacterial action of cultures of a Penicillium, with special reference to their use in the isolation of *B. influenzæ*. *Br. J. Exp. Pathol.* **10**, 226–236 (1929). PMC2566493 11545337

[3] Schatz, A., Bugie, E. & Waksman, S. Streptomycin: a substance exhibiting antibiotic activity against Gram-positive and Gram-negative bacteria. *Proc. Soc. Exp. Biol. Med.* **55**, 66–69 (1944). 10.1097/01.blo.0000175887.98112.fe 16056018

[4] Waksman, S. A., Schatz, A. & Reynolds, D. M. Production of antibiotic substances by Actinomycetes. *Ann. N. Y. Acad. Sci.* **1213**, 112–124 (2010). 21175680 10.1111/j.1749-6632.2010.05861.x

[5] Cox, G. et al. A common platform for antibiotic dereplication and adjuvant discovery. *Cell Chem. Biol.* **24**, 98–109 (2017). 28017602 10.1016/j.chembiol.2016.11.011

[6] Newman, D. J. Old and modern antibiotic structures with potential for today’s infections. *ADMET DMPK* **10**, 131–146 (2022). 35350115 10.5599/admet.1272 PMC8957243

[7] Bérdy, J. Thoughts and facts about antibiotics: where we are now and where we are heading. *J. Antibiot. (Tokyo)* **65**, 385–395 (2012). 22511224 10.1038/ja.2012.27

[8] Kirst, H. A. Developing new antibacterials through natural product research. *Expert Opin. Drug Discov.* **8**, 479–493 (2013). 23480029 10.1517/17460441.2013.779666

[9] Bartlett, J. G., Gilbert, D. N. & Spellberg, B. Seven ways to preserve the miracle of antibiotics. *Clin. Infect. Dis.* **56**, 1445–1450 (2013). 23403172 10.1093/cid/cit070

[10] Stokes, J. M. et al. A deep learning approach to antibiotic discovery. *Cell* **180**, 688–702.e13 (2020). 32084340 10.1016/j.cell.2020.01.021 PMC8349178

[11] Watve, M. G., Tickoo, R., Jog, M. M. & Bhole, B. D. How many antibiotics are produced by the genus Streptomyces? *Arch. Microbiol.* **176**, 386–390 (2001). 11702082 10.1007/s002030100345

[12] Ait, B. E. et al. Taxonomy, physiology, and natural products of actinobacteria. Microbiol. *Mol. Biol. Rev*. **80**, 1–43 (2015). 10.1128/MMBR.00019-15 PMC4711186 26609051

[13] Linares, J. F., Gustafsson, I., Baquero, F. & Martinez, J. L. Antibiotics as intermicrobial signaling agents instead of weapons. *Proc. Natl. Acad. Sci. USA* **103**, 19484–19489 (2006). 17148599 10.1073/pnas.0608949103 PMC1682013

[14] Meyer, K. J. & Nodwell, J. R. Biology and applications of co-produced, synergistic antimicrobials from environmental bacteria. *Nat. Microbiol.* **6**, 1118–1128 (2021). 34446927 10.1038/s41564-021-00952-6

[15] Hoskisson P. A. & Seipke R. F. Cryptic or silent? The known unknowns, unknown knowns, and unknown unknowns of secondary metabolism. *mBio* **11**, 10.1128/mbio.02642-20 (2020). 10.1128/mBio.02642-20 PMC7587438 33082252

[16] Blin, K. et al. antiSMASH 7.0: new and improved predictions for detection, regulation, chemical structures and visualisation. *Nucleic Acids Res.* **51**, W46–W50 (2023). 37140036 10.1093/nar/gkad344 PMC10320115

[17] Viaene, T., Langendries, S., Beirinckx, S., Maes, M. & Goormachtig, S. Streptomyces as a plant’s best friend? *FEMS Microbiol. Ecol*. **92**, fiw119 (2016). 27279415 10.1093/femsec/fiw119

[18] Batey, S. F. D., Greco, C., Hutchings, M. I. & Wilkinson, B. Chemical warfare between fungus-growing ants and their pathogens. *Mech. Biol. Energy* **59**, 172–181 (2020). 10.1016/j.cbpa.2020.08.001 PMC7763482 32949983

[19] Xu, H.-F. et al. Reading and surviving the harsh conditions in desert biological soil crust: the cyanobacterial viewpoint. *FEMS Microbiol. Rev.* **45**, fuab036 (2021). 34165541 10.1093/femsre/fuab036

[20] Xie, F. & Pathom-Aree, W. Actinobacteria from desert: diversity and biotechnological applications. *Front. Microbiol.* **12**, 765531 (2021). 34956128 10.3389/fmicb.2021.765531 PMC8696123

[21] Schulze-Makuch, D. et al. Transitory microbial habitat in the hyperarid Atacama Desert. *Proc. Natl. Acad. Sci. USA* **115**, 2670–2675 (2018). 29483268 10.1073/pnas.1714341115 PMC5856521

[22] Rateb, M. E., Ebel, R. & Jaspars, M. Natural product diversity of actinobacteria in the Atacama Desert. *Antonie Van Leeuwenhoek* **111**, 1467–1477 (2018). 29445902 10.1007/s10482-018-1030-z

[23] Mohammadipanah, F. & Wink, J. Actinobacteria from arid and desert habitats: diversity and biological activity. *Front. Microbiol.* **6**, 1541 (2015). 26858692 10.3389/fmicb.2015.01541 PMC4729944

[24] Ivanova, V. et al. Structural elucidation of a bioactive metabolites produced by streptomyces avidinii sb9 strain, isolated from permafrost soil in spitsbergen, arctic. *Biotechnol. Biotechnol. Equip.* **24**, 2092–2095 (2010).

[25] Moon, K. et al. New benzoxazine secondary metabolites from an arctic actinomycete. *Mar. Drugs* **12**, 2526–2538 (2014). 24796308 10.3390/md12052526 PMC4052304

[26] Wu, B. et al. Lindgomycin, an UNusual Antibiotic Polyketide from A Marine Fungus of the Lindgomycetaceae. *Mar. Drugs* **13**, 4617–4632 (2015). 26225984 10.3390/md13084617 PMC4556996

[27] Corral, P. et al. Identification of a sorbicillinoid-producing aspergillus strain with antimicrobial activity against Staphylococcus aureus: a new polyextremophilic marine fungus from barents sea. *Mar. Biotechnol. N. Y. N* **20**, 502–511 (2018). 10.1007/s10126-018-9821-9 29651633

[28] Núñez-Montero, K. & Barrientos, L. Advances in Antarctic research for antimicrobial discovery: a comprehensive narrative review of bacteria from antarctic environments as potential sources of novel antibiotic compounds against human pathogens and microorganisms of industrial importance. *Antibiot. Basel Switz* **7**, 90 (2018). 10.3390/antibiotics7040090 PMC6316688 30347637

[29] Tortorella, E. et al. Antibiotics from deep-sea microorganisms: current discoveries and perspectives. *Mar. Drugs* **16**, 355 (2018). 30274274 10.3390/md16100355 PMC6213577

[30] Cong, M. et al. Deep-sea natural products from extreme environments: cold seeps and hydrothermal Vents. *Mar. Drugs* **20**, 404 (2022). 35736207 10.3390/md20060404 PMC9229347

[31] Wang, C., Lu, Y. & Cao, S. Antimicrobial compounds from marine actinomycetes. *Arch. Pharm. Res.* **43**, 677–704 (2020). 32691395 10.1007/s12272-020-01251-0 PMC7703873

[32] Chen, J., Xu, L., Zhou, Y. & Han, B. Natural products from actinomycetes associated with marine organisms. *Mar. Drugs* **19**, 629 (2021). 34822500 10.3390/md19110629 PMC8621598

[33] Han, Y. et al. A comprehensive genomic catalog from global cold seeps. *Sci. Data* **10**, 596 (2023). 37684262 10.1038/s41597-023-02521-4 PMC10491686

[34] Wang, L. et al. Metagenomic insights into the functions of microbial communities in sulfur-rich sediment of a shallow-water hydrothermal vent off Kueishan Island. *Front. Microbiol.* **13**, 992034 (2022). 36532441 10.3389/fmicb.2022.992034 PMC9748435

[35] Shi, Y. et al. Stress-driven discovery of a cryptic antibiotic produced by Streptomyces sp. WU20 from Kueishantao hydrothermal vent with an integrated metabolomics strategy. *Appl. Microbiol. Biotechnol.* **101**, 1395–1408 (2017). 27730337 10.1007/s00253-016-7823-y

[36] Rothschild, L. J. & Mancinelli, R. L. Life in extreme environments. *Nature* **409**, 1092–1101 (2001). 11234023 10.1038/35059215

[37] O’Connor, E. M. & Shand, R. F. Halocins and sulfolobicins: the emerging story of archaeal protein and peptide antibiotics. *J. Ind. Microbiol. Biotechnol.* **28**, 23–31 (2002). 11938468 10.1038/sj/jim/7000190

[38] Virués-Segovia, J. R. et al. Kribbellichelins A and B, two new antibiotics from Kribbella sp. CA-293567 with activity against several human pathogens. *Molecules* **27**, 6355 (2022). 36234892 10.3390/molecules27196355 PMC9570599

[39] Lewis, K., Epstein, S., D’Onofrio, A. & Ling, L. L. Uncultured microorganisms as a source of secondary metabolites. *J. Antibiot. (Tokyo)* **63**, 468–476 (2010). 20648021 10.1038/ja.2010.87

[40] Baranova, A. A., Alferova, V. A., Korshun, V. A. & Tyurin, A. P. Modern trends in natural antibiotic discovery. *Life* **13**, 1073 (2023). 37240718 10.3390/life13051073 PMC10221674

[41] Nichols, D. et al. Use of Ichip for high-throughput in situ cultivation of “uncultivable” microbial species. *Appl. Environ. Microbiol.* **76**, 2445–2450 (2010). 20173072 10.1128/AEM.01754-09 PMC2849220

[42] Piddock, L. J. V. Teixobactin, the first of a new class of antibiotics discovered by iChip technology? *J. Antimicrob. Chemother.* **70**, 2679–2680 (2015). 26089440 10.1093/jac/dkv175

[43] Shukla, R. et al. An antibiotic from an uncultured bacterium binds to an immutable target. *Cell* **186**, 4059–4073.e27 (2023). 37611581 10.1016/j.cell.2023.07.038

[44] Hegemann, J. D., Birkelbach, J., Walesch, S. & Müller, R. Current developments in antibiotic discovery. *EMBO Rep.* **24**, e56184 (2023). 36541849 10.15252/embr.202256184 PMC9827545

[45] Hoffmann, T. et al. Correlating chemical diversity with taxonomic distance for discovery of natural products in myxobacteria. *Nat. Commun.* **9**, 803 (2018). 29476047 10.1038/s41467-018-03184-1 PMC5824889

[46] Zheng, S. et al. Synthesis and fungicidal activity of tryptophan analogues—the unexpected calycanthaceous alkaloid derivatives. *Nat. Prod. Res.* **31**, 1142–1149 (2017). 27653454 10.1080/14786419.2016.1230117

[47] Bassett, E. J., Keith, M. S., Armelagos, G. J., Martin, D. L. & Villanueva, A. R. Tetracycline-labeled human bone from ancient Sudanese Nubia (A.D. 350). *Science* **209**, 1532–1534 (1980). 7001623 10.1126/science.7001623

[48] Nelson, M. L. & Levy, S. B. The history of the tetracyclines. *Ann. N. Y. Acad. Sci.* **1241**, 17–32 (2011). 22191524 10.1111/j.1749-6632.2011.06354.x

[49] Maciejewska, M. et al. A phenotypic and genotypic analysis of the antimicrobial potential of cultivable streptomyces isolated from cave moonmilk deposits. *Front. Microbiol.* **7**, 1455 (2016). 27708627 10.3389/fmicb.2016.01455 PMC5030222

[50] Rangseekaew, P. & Pathom-aree, W. Cave actinobacteria as producers of bioactive metabolites. *Front. Microbiol.* **10**, 387 (2019). 30967844 10.3389/fmicb.2019.00387 PMC6438885

[51] Falkinham, J. O. 3rd et al. Proliferation of antibiotic-producing bacteria and concomitant antibiotic production as the basis for the antibiotic activity of Jordan’s red soils. *Appl. Environ. Microbiol.* **75**, 2735–2741 (2009). 19286796 10.1128/AEM.00104-09 PMC2681674

[52] Terra, L. et al. A novel alkaliphilic Streptomyces inhibits ESKAPE pathogens. *Front. Microbiol.* **9**, 2458 (2018). 30459722 10.3389/fmicb.2018.02458 PMC6232825

[53] Behroozian, S., Svensson, S. L. & Davies, J. Kisameet clay exhibits potent antibacterial activity against the ESKAPE pathogens. *mBio* **7**, e01842–15 (2016). 26814180 10.1128/mBio.01842-15 PMC4742703

[54] Quinn, G. A. et al. Streptomyces Isolates from the Soil of an Ancient Irish Cure Site, Capable of Inhibiting Multi-Resistant Bacteria and Yeasts. *Appl. Sci.* **11**, 4923 (2021).

[55] Cordell, et al. In: *Anticancer Drug Discovery and Development: Natural Products and New Molecular Models. Developments in Oncology*. (Springer, 1994)

[56] Harrison, F. et al. A 1000-year-old antimicrobial remedy with antistaphylococcal activity. *mBio* **6**, e01129–15 (2015). 26265721 10.1128/mBio.01129-15 PMC4542191

[57] Harrison, F., Blower, A., de Wolf, C. & Connelly, E. Sweet and sour synergy: exploring the antibacterial and antibiofilm activity of acetic acid and vinegar combined with medical-grade honeys. *Microbiology* **169**, 001351 (2023). 37435775 10.1099/mic.0.001351 PMC10433418

[58] Joshi-Navare, K. & Prabhune, A. A Biosurfactant-Sophorolipid Acts in Synergy with Antibiotics to Enhance Their Efficiency. *BioMed Res. Int.* **2013**, 512495 (2013). 24089681 10.1155/2013/512495 PMC3782141

[59] Al-Healy, N. H. & Al-Sammak, E. Gh. effects of *Streptomyces rochi* biosurfactants on pathogenic *Staphylococcus aureus* and *Pseudomonas aeruginosa*. *Al-Mukhtar J. Sci*. **37**, 261–273 (2022).

[60] Rabahi, M. F., Silva Júnior, J. L. R., da, Ferreira, A. C. G., Tannus-Silva, D. G. S. & Conde, M. B. Tuberculosis treatment. *J. Bras. Pneumol.* **43**, 472–486 (2017). 29340497 10.1590/S1806-37562016000000388 PMC5792048

[61] Mattingly, A. E. et al. Screening an established natural product library identifies secondary metabolites that potentiate conventional antibiotics. *ACS Infect. Dis.* **6**, 2629–2640 (2020). 32810395 10.1021/acsinfecdis.0c00259 PMC8330956

[62] Nikolaev, Y. A. et al. The use of 4-hexylresorcinol as antibiotic adjuvant. *PLoS ONE* **15**, e0239147 (2020). 32960928 10.1371/journal.pone.0239147 PMC7508414

[63] van Asbeck, B. S., Marcelis, J. H., van Kats, J. H., Jaarsma, E. Y. & Verhoef, J. Synergy between the iron chelator deferoxamine and the antimicrobial agents gentamicin, chloramphenicol, cefalothin, cefotiam and cefsulodin. *Eur. J. Clin. Microbiol* **2**, 432–438 (1983). 6315421 10.1007/BF02013900

[64] Terra, L., Dyson, P., Ratcliffe, N., Castro, H. C. & Vicente, A. C. P. Biotechnological potential of Streptomyces siderophores as new antibiotics. *Curr. Med. Chem.*10.2174/0929867327666200510235512 (2020). 10.2174/0929867327666200510235512 32389112

[65] Nüesch, J. & Knüsel, F. in *Mech. Action* (eds. Gottlieb, D. & Shaw, P. D.) 499–541 (Springer Berlin Heidelberg, 1967).

[66] Wang, M., Zhang, Y., Lv, L., Kong, D. & Niu, G. Biosynthesis and chemical synthesis of albomycin nucleoside antibiotics. *Antibiotics* **11**, 438 (2022). 35453190 10.3390/antibiotics11040438 PMC9032320

[67] Sackmann, W. et al. a new iron-containing antibiotic. *Antibiot. Chemother. Northfield Ill* **12**, 34–45 (1962). 14037612

[68] Ibrahim, W. M. et al. Exploring the antimicrobial, antiviral, antioxidant, and antitumor potentials of marine Streptomyces tunisiensis W4MT573222 pigment isolated from Abu-Qir sediments, Egypt. *Microb. Cell Factories* **22**, 94 (2023). 10.1186/s12934-023-02106-1 PMC10161460 37147660

[69] Abraham, J. & Chauhan, R. Profiling of red pigment produced by Streptomyces sp. JAR6 and its bioactivity. *3 Biotech* **8**, 22 (2017). 29276660 10.1007/s13205-017-1044-7 PMC5735041

[70] Chen X et al. Genome sequence of *Streptomyces violaceusniger* Strain SPC6, a halotolerant Streptomycete that exhibits rapid growth and development. *Genome Announc*. **1**, 10.1128/genomea.00494-13 (2013). 10.1128/genomeA.00494-13 PMC3715669 23868127

[71] Chen, X. et al. A new bacterial tRNA enhances antibiotic production in Streptomyces by circumventing inefficient wobble base-pairing. *Nucleic Acids Res.* **50**, 7084–7096 (2022). 35699212 10.1093/nar/gkac502 PMC9262613

[72] Zhang, L. et al. Characterization of anti-BCG benz[α]anthraquinones and new siderophores from a Xinjiang desert-isolated rare actinomycete Nocardia sp. XJ31. *Appl. Microbiol. Biotechnol.* **104**, 8267–8278 (2020). 32830291 10.1007/s00253-020-10842-2 PMC7443361

[73] Merrouche, R. et al. A new dithiolopyrrolone antibiotic triggered by a long fermentation of *Saccharothrix algeriensis* NRRL B-24137 in sorbic acid-amended medium. *Lett. Appl. Microbiol.* **69**, 294–301 (2019). 31424588 10.1111/lam.13207

[74] Abdelkader, M. S. A. et al. Asenjonamides A–C, antibacterial metabolites isolated from Streptomyces asenjonii strain KNN 42.f from an extreme-hyper arid Atacama Desert soil. *J. Antibiot. (Tokyo)* **71**, 425–431 (2018). 29362461 10.1038/s41429-017-0012-0

[75] Rateb, M. E. et al. Diverse metabolic profiles of a Streptomyces strain isolated from a hyper-arid environment. *J. Nat. Prod.* **74**, 1965–1971 (2011). 21879726 10.1021/np200470u

[76] Rateb, M. E. et al. Chaxamycins A–D, bioactive ansamycins from a hyper-arid desert Streptomyces sp. *J. Nat. Prod.* **74**, 1491–1499 (2011). 21553813 10.1021/np200320u

[77] Nachtigall, J. et al. Atacamycins A–C, 22-membered antitumor macrolactones produced by Streptomyces sp. C38. *J. Antibiot. (Tokyo)* **64**, 775–780 (2011). 22008702 10.1038/ja.2011.96

[78] Schulz, D. et al. Abenquines A–D: aminoquinone derivatives produced by Streptomyces sp. strain DB634. *J. Antibiot. (Tokyo)* **64**, 763–768 (2011). 21952099 10.1038/ja.2011.87

[79] Kavitha, A., Prabhakar, P., Vijayalakshmi, M. & Venkateswarlu, Y. Purification and biological evaluation of the metabolites produced by Streptomyces sp. TK-VL_333. *Res. Microbiol.* **161**, 335–345 (2010). 20403429 10.1016/j.resmic.2010.03.011

[80] Masand, M. et al. Biosynthetic potential of bioactive streptomycetes isolated from arid region of the Thar Desert, Rajasthan (India). *Front. Microbiol.* **9**, 687 (2018). 29720968 10.3389/fmicb.2018.00687 PMC5915549

[81] Chi, L.-P., Li, X.-M., Wan, Y.-P., Li, X. & Wang, B.-G. Ophiobolin sesterterpenoids and farnesylated phthalide derivatives from the deep sea cold-seep-derived fungus Aspergillus insuetus SD-512. *J. Nat. Prod.* **83**, 3652–3660 (2020). 33322904 10.1021/acs.jnatprod.0c00860

[82] Jin, E., Li, H., Liu, Z., Xiao, F. & Li, W. Antibiotic dixiamycins from a cold-seep-derived *Streptomyces olivaceus*. *J. Nat. Prod.* **84**, 2606–2611 (2021). 34410142 10.1021/acs.jnatprod.1c00411

[83] Lü, F., Li, X., Chi, L., Meng, L. & Wang, B. A new acyclic peroxide from *Aspergillus nidulans* SD-531, a fungus obtained from deep-sea sediment of cold spring in the South China Sea. *J. Oceanol. Limnol* **38**, 1225–1232 (2020).

[84] Zhou, X. et al. Marthiapeptide A, an anti-infective and cytotoxic polythiazole cyclopeptide from a 60 L scale fermentation of the deep sea-derived *Marinactinospora thermotolerans* SCSIO 00652. *J. Nat. Prod.* **75**, 2251–2255 (2012). 23215246 10.1021/np300554f

[85] Song, Y. et al. Cyclic hexapeptides from the deep south china sea-derived *Streptomyces scopuliridis* SCSIO ZJ46 active against pathogenic Gram-positive bacteria. *J. Nat. Prod.* **77**, 1937–1941 (2014). 25072108 10.1021/np500399v

[86] Pérez-Bonilla, M. et al. Phocoenamicins B and C, new antibacterial spirotetronates isolated from a marine micromonospora sp. *Mar. Drugs* **16**, 95 (2018). 29547589 10.3390/md16030095 PMC5867639

[87] Choi, E. J., Kwon, H. C., Ham, J. & Yang, H. O. 6-Hydroxymethyl-1-phenazine-carboxamide and 1,6-phenazinedimethanol from a marine bacterium, Brevibacterium sp. KMD 003, associated with marine purple vase sponge. *J. Antibiot. (Tokyo)* **62**, 621–624 (2009). 19798118 10.1038/ja.2009.92

[88] Vicente, J. et al. Monacyclinones, new angucyclinone metabolites isolated from Streptomyces sp. M7_15 associated with the Puerto Rican sponge Scopalina ruetzleri. *Mar. Drugs* **13**, 4682–4700 (2015). 26230704 10.3390/md13084682 PMC4556999

[89] Tawfike, A. et al. New bioactive metabolites from the elicited marine sponge-derived bacterium Actinokineospora spheciospongiae sp. nov. *AMB Express* **9**, 12 (2019). 30680548 10.1186/s13568-018-0730-0 PMC6345950

[90] Cheng, C. et al. Ageloline A, new antioxidant and antichlamydial quinolone from the marine sponge-derived bacterium Streptomyces sp. SBT345. *Tetrahedron Lett*. **57**, 2786–2789 (2016).

